# Real-Time Adaptive Modulation Schemes for Underwater Acoustic OFDM Communication

**DOI:** 10.3390/s22093436

**Published:** 2022-04-30

**Authors:** Suchi Barua, Yue Rong, Sven Nordholm, Peng Chen

**Affiliations:** School of Electrical Engineering, Computing and Mathematical Sciences (EECMS), Faculty of Science and Engineering, Curtin University, Bentley, WA 6102, Australia; suchi.barua@postgrad.curtin.edu.au (S.B.); s.nordholm@curtin.edu.au (S.N.); peng.chen@curtin.edu.au (P.C.)

**Keywords:** underwater acoustic communication, time-varying channel, orthogonal frequency-division multiplexing (OFDM), adaptive modulation, LabVIEW, CompactDAQ

## Abstract

Adaptive modulation received significant attention for underwater acoustic (UA) communication systems with the aim of increasing the system efficiency. It is challenging to attain a high data rate in UA communication, as UA channels vary fast, along with the environmental factors. For a time-varying UA channel, a self-adaptive system is an attractive option, which can choose the best method according to the channel condition to guarantee the continuous connectivity and high performance constantly. A real-time orthogonal frequency-division multiplexing (OFDM)-based adaptive UA communication system is presented in this paper, employing the National Instruments (NI) LabVIEW software and NI CompactDAQ device. In this paper, the received SNR is considered as a performance metric to select the transmission parameters, which are sent back to the transmitter for data transmission. In this research, a UA OFDM communication system is developed, employing adaptive modulation schemes for a nonstationary UA environment which allows to select subcarriers, modulation size, and allocate power adaptively to enhance the reliability of communication, guarantee continuous connectivity, and boost data rate. The recent UA communication experiments carried out in the Canning River, Western Australia, verify the performance of the proposed adaptive UA OFDM system, and the experimental results confirm the superiority of the proposed adaptive scheme.

## 1. Introduction

Underwater acoustic (UA) wireless communication plays an important role in exploring the marine environment, including observation of marine life and water pollution, monitoring of subsea infrastructure such as oil rigs and pipelines, surveillance of natural disasters, climate change prediction, and naval tactical operations for coastal securities [1,2]. High-speed communication in the UA channel is vitally important for marine commercial operations, offshore oil and gas industries, and defence applications. These applications require reliable and vigorous underwater communication systems to be employed to establish a sophisticated underwater network [3,4]. 

High-data-rate communication is difficult in UA communication, as UA channels vary fast according to environmental circumstances. Rapid time variation, refractive properties of the medium, randomly varying multi-path propagation, severe fading due to limited bandwidth, and large Doppler shift due to motion are the main constraints of the UA channel. That is why the UA channel is known as one of the most demanding channels for reliable communication [5]. Orthogonal frequency-division multiplexing (OFDM) has become an attractive choice for UA communication because it remarkably mitigates the multipath interference with a low computational complexity [5,6,7]. The objective of the research is to develop the self-adapting algorithms by analysing the channel characteristics and varying transmission parameters, depending on the channel condition. 

In fixed (non-adaptive) modulation, the transmitter does not have any information about the channel conditions to exploit in choosing the transmission parameters. On the other hand, in the adaptive modulation system, the receiver feeds back the knowledge of the channel information to the transmitter, and the performance of an adaptive modulation scheme relies on the transmitter’s knowledge of the channel [8,9,10]. In this research, adaptive modulation is considered with the aim of attaining high spectral efficiency, enhancing the reliability of communication, improving the energy efficiency, and boosting the data rate. Moreover, cluster-based adaptive modulation is chosen in this research to minimize the feedback load and computational complexity of the adaptive scheme [9]. 

The estimated received signal-to-noise ratio (SNR) and the received cluster SNR are used as the channel state information (CSI) for the frame-based and the cluster-based adaptive modulation systems, respectively. Then, the target SNR and the SNR thresholds are determined. After that, depending on the thresholds, the transmission parameters are adaptively chosen for each frame/cluster and sent back to the transmitter for the next transmission. In [11,12], the proposed frame-based adaptive scheme enables the system to adaptively allocate the modulation size for each frame. The performance of the frame-based adaptive modulation scheme is verified with the simulation and tank experiment results in [11,12]. In [13], a cluster-based adaptive modulation is proposed, where the system adaptively allocates the modulation size for each cluster. However, in this paper, the proposed cluster-based adaptive scheme enables the system to adaptively allocate the modulation size as well as the data subcarriers and distribute the power on the data subcarriers for the next transmission. In this paper, the real-time river experiment results for the cluster-based modulation scheme, which is carried out in the Canning River, Western Australia, are also presented. The experimental results confirm the superiority of the proposed adaptive scheme. 

The proposed cluster-based adaptive modulation scheme allows the system:

- To choose a proper modulation, depending on the channel conditions, to improve the system throughput under a fixed bit error rate (BER).
- To discard subcarriers that experience deep fade to improve the energy efficiency of the system. Discarding the deep-faded (weak) subcarriers helps to save the energy which is wasted to transmit the deep-faded subcarriers, and this energy is distributed on the remaining subcarriers for the next transmission.
- To distribute the residual power among the remaining subcarriers, which ensures constant transmitted symbol energy, despite the channel variation, to achieve overall better throughput of the system.

In this research, a combination of the National Instruments (NI) CompactDAQ device and NI LabVIEW software are adopted for real-time adaptive modulation for UA OFDM communication. The NI-based implementation offers simplified integration with the hardware and provides rapid data acquisition and visualization from NI input/output (I/O) devices or third-party I/O devices. These features of NI-based implementation make the system flexible, help to simplify the prototype design, and keep the software development time short compared to digital signal processor (DSP)-based design [14,15].

## 2. Background

Long-range and high-data-rate communication in the UA channel is vitally important for marine commercial operations, offshore oil and gas industries, and defence applications. In underwater communications, acoustic waves are preferred over electromagnetic waves, as the latter suffers from high attenuation and severe scattering in the medium of water. However, the UA channel is extremely bandlimited and fast-varying spatially and temporally [16,17,18], which poses many obstacles to enhance the data rate of UA communication systems over a long distance [4,19,20].

Remarkable progresses have been made in single-carrier communication systems in UA communication [21,22]. However, in recent years, OFDM has been very actively pursued for UA communications because of its resilience to frequency-selective channels with long delay spreads [5]. The complexity in designing the receiver is lower in OFDM than the single carrier systems. By dividing the available bandwidth into a number of narrower bands, the OFDM system can perform equalization in the frequency domain, thus eliminating the need for complex time-domain equalizers. In recent years, adaptive modulation has been appealing to UA communication systems to achieve a high bandwidth efficiency by exploiting knowledge of the channel state. This is particularly effective in the UA scenario, where high spectral efficiencies are critical to achieve, since available bandwidth is extremely limited due to frequency-dependent attenuation of the UA channel [8,9,10].

It can be noted that despite recent progresses in UA OFDM communications, most of the published research outcomes are based on either numerical simulations or offline signal processing of recorded data. Regarding UA OFDM modem development, there are limited works which are published in [23,24,25]. Even fewer work has been published on adaptive modulation in UA OFDM communication [8,9,10,26,27] which considers real-time data. Unfortunately, using simulations or offline processing may underestimate the challenges in real-time high-speed UA communication systems. In practice, the computation complexity of signal processing algorithms for UA communication increases dramatically due to the increasing inter-symbol interference (ISI) and Doppler effect, which makes real-time communication more challenging. In this research, real-time adaptive modulation for UA OFDM communication has been considered and implemented for different adaptive schemes. In Section 6.1.2 and Section 6.2.1 of this paper, experimental results are presented and discussed, which are performed in a tank and Canning River, Western Australia.

In [24,25,28], the TMS320C6713 DSP development board has been used to implement an OFDM-based modem for UA communication. However, DSP and field-programmable gate array (FPGA)-based implementation can be time-consuming on system design and development [29]. In [14,15], a real-time OFDM-based UA communication system was implemented using the NI CompactDAQ device and the LabVIEW software. Compared with FPGA and DSP-based designs, the NI CompactDAQ device combined with the LabVIEW software is more flexible, makes the prototype design simpler, and reduces the software development time [14,15]. Generally, a detailed knowledge of the specific hardware and/or software architecture is required to modify a UA modem that is implemented on a dedicated architecture such as DSP or FPGA. In contrast, there is less specialized knowledge needed on this system as it is running on general purpose processors (GPPs). The proposed system does not require to program a dedicated processor, such as DSP, which is a relief for the researchers and allows them to spend the time in developing high-performance UA communication algorithms. Moreover, the LabVIEW programming environment offers simplified hardware integration, which permits a fast system configuration.

## 3. System Model

**Figure 1:**
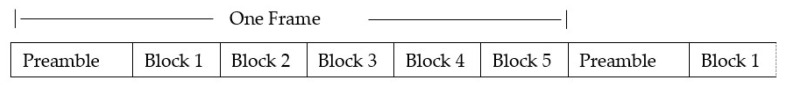
Frame structure of the UA OFDM system.

A frame-based UA OFDM communication system is considered in this research. Figure 1 shows the frame structure of the transmitted signals. Each frame consists of one preamble block and five OFDM data blocks. The preamble block is used for synchronization and each data block consists of data subcarriers, pilot subcarriers, and null subcarriers. It is assumed that pilot subcarriers are uniformly spaced [6,7,14,15]. In this system, different types of subcarriers are used in a frame for different purposes.

- Pilot subcarriers are used for channel estimation.
- Data subcarriers are used to carry information symbols.
- Null subcarriers are used at the edge of the frequency band to prevent spectrum leakage. Null subcarriers are also placed among the active subcarriers (pilot and data subcarriers) to facilitate Doppler estimation and noise variance estimation.

Let us introduce $K_{p}$ as the number of pilot subcarriers, $K_{d}$ as the number of data subcarriers, and $K_{n}$ as the number of null subcarriers. In each frame, a binary source data stream b is generated:

(1) $$b={\left(b\left[1\right],\ldots ,b\left[K_{b}\right]\right)}^{T}$$

where ${\left(\cdot \right)}^{T}$ denotes the matrix (vector) transpose, $K_{b}=MK_{d}K_{blk}$ is the number of information-carrying bits in each frame, M denotes the modulation order (e.g., 1 for BPSK, 2 for QPSK, and 4 for 16QAM), and $K_{blk}$ denotes the number of OFDM blocks in one frame.

Then, the OFDM symbol vector s is mapped from b, depending on modulation constellations

(2) $$s={\left(s\left[1\right],\ldots ,s\left[K_{s}\right]\right)}^{T}$$

where $K_{s}=K_{p}+K_{d}+K_{n}$ is the number of total subcarriers. Each OFDM symbol is converted to the time domain by the inverse discrete Fourier transform (IDFT), leading to the following baseband discrete time signal:

(3) $$x=F^{H}s$$

where ${\left(\cdot \right)}^{H}$ denotes the conjugate transpose and F is a $K_{s}$ × $K_{s}$ discrete Fourier transform (DFT) matrix with the $\left(i,k\right)$-th entry of $1/\sqrt{K_{s}}e^{-j2\pi \left(i-1\right)\left(k-1\right)/K_{s}}$, $i,k=1,\ldots ,\;K_{s}$. Passband signals are directly generated for each OFDM block at the transmitter. The bandwidth of the transmitted signal is $B=f_{sc}K_{s}$, where $f_{sc}$ is the subcarrier spacing. The duration of one OFDM symbol is $T=1/f_{sc}$. The $K_{s}$ subcarriers are located at frequencies of

$$f_{k}=f_{c}+k\;f_{sc},\;\;\;\;\;\;k=-\frac{K_{s}}{2}+1,\ldots ,\;\frac{K_{s}}{2}$$

where $f_{c}$ is the center frequency. To avoid interference among OFDM blocks, a cyclic prefix (CP) of length $T_{cp}$ is prepended to the OFDM symbol, and the total length of one OFDM block is $T_{total}=T+T_{cp}$. The continuous time representation of an OFDM block can be expressed as

$$\tilde{x}\left(t\right)=2\mathbb{R}\left\{\left[\frac{1}{\sqrt{K_{s}}}\overset{\frac{K_{s}}{2}}{\underset{-\frac{K_{s}}{2}+1}{\sum }}\overset{ˇ}{s}\left[k\right]e^{j2\pi kf_{sc}t}\right]\;e^{j2\pi f_{c}t}\right\}\;,\;\;0\;\leq \;t\;\leq \;T$$

(4) $$\tilde{x}\left(t\right)=\tilde{x}\left(t+T\right),\;\;\;\;\;\;\;\;\;\;\;\;\;\;\;\;\;\;\;\;\;\;\;\;\;\;\;\;-T_{cp}\;\leq \;t\;<\;0$$

where $\mathbb{R}\;\left\{\cdot \right\}$ denotes the real part of a complex number and

$$\overset{ˇ}{s}\left[k\right]=\left\{\begin{array}{cc} s\left[k\right], & 1\leq k\leq \frac{K_{s}}{2}\\ s\left[k+K_{s}\right], & -\frac{K_{s}}{2}+1\leq k\leq 0 \end{array}\right.$$

A general UA channel with $N_{p}$ paths can be represented as

(5) $$h\left(t,\tau \right)=\overset{N_{p}}{\underset{p=1}{\sum }}A_{p}\left(t\right)\delta \left(t-\tau_{p}\left(t\right)\right)$$

where $N_{p}$ is the number of propagation path, $\delta \left(.\right)$ is the Dirac delta function, and t is the time at which the channel is observed. The coefficient $A_{p}\left(t\right)$ and $\tau_{p}\left(t\right)$ represent the amplitude and delay of the pth path, respectively [6,30]. 

In general, UA communication suffers from time-varying frequency offset caused by the change of $\tau_{p}\left(t\right)$ within one OFDM block. In the following, we assume that $A_{p}\left(t\right)$ is constant and all paths have the same Doppler scaling factor ϵ during one OFDM block. Thus,

$$\tau_{p}\left(t\right)\approx \tau_{p}-\epsilon t,\;\;\;\;\;\;\;p=1,\;\ldots ,\;N_{p}.$$

Then the received passband signal of one OFDM block is given by

(6) $$\tilde{r}\left(t\right)=\sum_{p=1}^{N_{p}}A_{p}\;\tilde{x}\left(t-\tau_{p}+\epsilon t\right)+\tilde{w}\left(t\right).$$

where $\tilde{w}\left(t\right)$ is the passband additive noise. Then the Doppler scaling factor $\overset{ˇ}{\epsilon }$ is estimated and used to resample the received signal. After removing the CP, downshifting, and low-pass filtering $\tilde{r}\left(t\right)$, the baseband received signal can be obtained from Equations (4) and (6), as

$$r\left(t\right)\approx e^{j2\pi \;\overset{ˇ}{\epsilon }t}\overset{N_{p}}{\underset{p=1}{\sum }}\frac{A_{p}e^{-j2\pi f_{c}\tau_{p}}}{\sqrt{K_{s}}}\;\;\overset{\frac{K_{s}}{2}}{\underset{k=-\frac{K_{s}}{2}+1}{\sum }}\overset{ˇ}{s}\left[k\right]e^{j2\pi kf_{sc}\left(t-\tau_{p}\right)}+w\left(t\right)$$

(7) $$=e^{j2\pi \;\overset{ˇ}{\epsilon }t}\frac{1}{\sqrt{K_{s}}}\;\overset{\frac{K_{s}}{2}}{\underset{k=-\frac{K_{s}}{2}+1}{\sum }}\overset{ˇ}{s}\left[k\right]e^{j2\pi kf_{sc}t}\overset{N_{p}}{\underset{p=1}{\sum }}A_{p}e^{-j2\pi f_{k}\tau_{p}}\;\;+w\left(t\right),\;\;0\leq t\leq T$$

where $\overset{ˇ}{\epsilon }$ represents the carrier frequency offset (CFO) introduced by the remaining Doppler shift, and $w\left(t\right)$ is the baseband additive noise. From Equation (7), the channel frequency response (CFR) at the k-th subcarrier is given by

(8) $$H\left[k\right]=\overset{N_{p}}{\underset{p=1}{\sum }}A_{p}e^{-j2\pi f_{k}\tau_{p}},\;\;\;\;\;\;k=-\frac{K_{s}}{2}+1,\;\ldots ,\frac{K_{s}}{2}$$

By sampling $r\left(t\right)$ at the rate of 1/B, we obtain discrete time samples of one OFDM symbol from Equation (7) as

$$r\left[i\right]=\frac{e^{j2\pi i\;\overset{ˇ}{\epsilon }/B}}{\sqrt{K_{s}}}\;\overset{\frac{K_{s}}{2}}{\underset{k=-\frac{K_{s}}{2}+1}{\sum }}\overset{ˇ}{s}\left[k\right]e^{j2\pi ikf_{sc}/B}\;H\left[k\right]+w\left[i\right]$$

(9) $$=\frac{e^{j2\pi i\;\overset{ˇ}{\epsilon }/B}}{\sqrt{K_{s}}}\;\overset{\frac{K_{s}}{2}}{\underset{k=-\frac{K_{s}}{2}+1}{\sum }}\overset{ˇ}{s}\left[k\right]e^{j2\pi ik/K_{s}}\;H\left[k\right]+w\left[i\right]\;i=1,\ldots ,\;K_{s}$$

where $w\left[i\right]$ is the noise sample. The matrix-vector form of Equation (9) is given by

(10) $$r_{t\;}=PF^{H}SFh_{t}+w_{t}$$

where

$$r_{t}={\left(r\left[1\right],\ldots r\left[K_{s}\right]\right)}^{T}$$

$$w_{t}={\left(w\left[1\right],\ldots w\left[K_{s}\right]\right)}^{T}$$

$$P=diag\left(1,\;e^{-j2\pi \;\overset{ˇ}{\epsilon }/B},\ldots ,e^{-j2\pi (K_{s}-1)\;\overset{ˇ}{\epsilon }/B}\right)$$

S = $diag\left(s\right)$ is a diagonal matrix taking s as the main diagonal elements, and $h_{t}$ is the discrete time domain representation of the channel impulse response (CIR) with a maximum delay of $N_{m}=\left\lceil B\tau_{N_{p}}\right\rceil$. After estimating and removing the CFO, which will be discussed in the next subsection, the frequency domain representation of the received signal is

(11) $$r_{f}=Sh_{f}+w_{f}$$

where $w_{f}$ is the additive noise vector in the frequency domain,

$$h_{f}={\left(h_{f}\left[1\right],\ldots .h_{f}\left[K_{s}\right]\right)}^{T}$$

is a vector containing the CFR at all $K_{s}$ subcarriers with

$$h_{f}\left[k\right]=\left\{\begin{array}{cc} H\left[k\right], & 1\leq k\leq \frac{K_{s}}{2}\\ H\left[k-K_{s}\right], & \frac{K_{s}}{2}+1\leq k\leq K_{s} \end{array}\right.$$

### 3.1. CFO Estimation

In underwater communications, the relative motion between a freely drifting transmitter and a receiver can reach a few metres per second even under the influence of mild wind and sea surface current. The platform motions and the medium instability lead to a fast-varying UA channel. Considering the slow velocity of sound in water, such motion generates strong Doppler effects. OFDM is sensitive to the motion-induced Doppler shift, which creates non-uniform frequency offset in a wideband acoustic signal [31]. The orthogonality relationship among the OFDM subcarriers is destroyed due to CFO and thus deteriorates the system performance severely. CFO estimation and compensation have vital importance for robust system performance.

In this research, it is assumed that the dominant Doppler shift is caused by the direct transmitter/receiver motion, and the channel has a common Doppler scaling factor on all propagation paths [32]. In general, different paths could have different Doppler scaling factors. When this is not the case, part of useful signals is treated as additive noise, which could considerably increase the overall noise variance, though it is found that, since the motion of the transmitter and/or receiver causes dominant Doppler shift, this assumption seems to be justified [33].

A two-step approach is used for CFO ϵ estimation which contains resampling and high-resolution uniform residual Doppler compensation [34,35]. At the receiver side, the CP is removed from the received signal and then CFO estimation is performed using null subcarrier in each OFDM block. The energy of the null subcarriers is used as the cost function as

(12) $$J\left(\epsilon \right)={\left|\Theta F\Gamma^{H}\left(\epsilon \right)r_{t}\right|}^{2}$$

where Θ is a selection matrix that picks the frequency-domain measurements on the null subcarriers out of all $K_{s}$ subcarriers, | | is the Euclidean norm of a vector,

$$\Gamma \left(\epsilon \right)=diag\left(1,e^{j2\pi T_{c}\epsilon },\ldots ,e^{j2\pi T_{c}(K_{s}-1)\epsilon }\right)$$

is diagonal matrix where $T_{c}=1/B$ is the time interval for each sample, and ${\left(.\right)}^{H}$ denotes the complex conjugate transpose operation of a matrix. An estimation of ϵ is found through

(13) $$\hat{\epsilon }=argmin_{\epsilon }\;J\left(\epsilon \right)$$

which is solved via 1D search on ϵ [34,35]. The CFO-induced inter-carrier interference (ICI) is greatly reduced after resampling and CFO compensation, and thus saves the system from performance degradation. An ICI-free reception can be almost achieved with this high-resolution algorithm that is similar to the MUSIC-like algorithm proposed in [34,36] for CP-OFDM.

### 3.2. Channel Estimation

UA channels have the characteristics of long and fast time-varying multipath delay; thus, estimating the channel is one of the technical challenges. As UA channels vary significantly in different environments and are doubly selective in both time and frequency [4], channel estimation is a key factor for the UA communication system performance.

Channel estimation is performed based on pilot subcarriers, which is the mainstream method at present. Least-squares estimation (LS) and minimum mean-squared-error estimation (MMSE) [37] are two major approaches for channel estimation. Then, the interpolation methods, such as linear interpolation or time-frequency conversion interpolation can be used for channel estimation. In our research, the LS method is used followed by linear interpolation for channel estimation.

After Doppler effect estimation and compensation, ideally, (11) can also be presented: as

(14) $$\left[\begin{array}{c} r_{f}\left[1\right]\\ r_{f}\left[2\right]\\ \vdots \\ r_{f}\left[K_{s}\right] \end{array}\right]=\left[\begin{array}{cccc} S\left[1\right] & 0 & \ldots & 0\\ 0 & S\left[2\right] & & \vdots \\ \vdots & & \ddots & 0\\ 0 & \ldots & 0 & S\left[K_{s}\right] \end{array}\right]\;\left[\begin{array}{c} h_{f}\left[1\right]\\ h_{f}\left[2\right]\\ \vdots \\ h_{f}\left[K_{s}\right] \end{array}\right]+\left[\begin{array}{c} w_{f}\left[1\right]\\ w_{f}\left[2\right]\\ \vdots \\ w_{f}\left[K_{s}\right] \end{array}\right]$$

We index pilot subcarriers as the set $K_{p}=\left\{p_{1},\ldots ,\;p_{K_{p}}\right\}$ with $K_{p}\;:={\bar{K}}_{p}$ subcarriers in total and for a set X, $\bar{X}$ denotes the number of elements in X. Based on the input–output relationship in Equation (14), arranging the frequency measurements at pilot subcarriers into a vector yields

(15) $$\left[\begin{array}{c} r_{f_{p}}\left[1\right]\\ r_{f_{p}}\left[2\right]\\ \vdots \\ r_{f_{p}}\left[K_{p}\right] \end{array}\right]=\left[\begin{array}{cccc} S_{p}\left[1\right] & 0 & \ldots & 0\\ 0 & S_{p}\left[2\right] & & \vdots \\ \vdots & & \ddots & 0\\ 0 & \ldots & 0 & S_{p}\left[K_{p}\right] \end{array}\right]\;\left[\begin{array}{c} h_{f_{p}}\left[1\right]\\ h_{f_{p}}\left[2\right]\\ \vdots \\ h_{f_{p}}\left[K_{p}\right] \end{array}\right]+\left[\begin{array}{c} w_{f_{p}}\left[1\right]\\ w_{f_{p}}\left[2\right]\\ \vdots \\ w_{f_{p}}\left[K_{p}\right] \end{array}\right]$$

Let us introduce $S_{p}=diag\left(S_{p}\left[1\right],\;S_{p}\left[2\right],\ldots S_{p}\left[K_{p}\right]\right)$ and $r_{p}$ as the received data in the pilot subcarriers in $r_{f}$ which are equally spaced within the $K_{s}$ subcarriers and are known to the receiver. In order to recover the data S, we need to estimate the CFR $h_{f}$. The LS-based channel estimation at pilot subcarriers is as follows [38,39]:

(16) $$\begin{array}{cc} {\hat{h}}_{LS} & ={\left(S_{p}^{H}\;S_{p}\right)}^{-1}\;\left(S_{p}^{H}\;r_{p}\right)\\ & ={S_{p}}^{-1}\;r_{p}. \end{array}$$

Then, linear interpolation is applied to obtain the channel responses at the data subcarriers based on the channel responses at the pilot subcarriers.

### 3.3. SNR Estimation

Limited bandwidth, strong multipath interference, severe fading, and significant Doppler shifts are the main limitations for UA communication. In addition to those challenges, UA noise has an impact on UA communication. UA noise is generated from different sources, such as turbulence, wind-generated waves, rain, surface shipping and industrial activities, marine animals, ice cracking, snapping shrimp, earthquakes at the seabed, oil and gas exploration and production, etc. [5,40].

Following the OFDM signal design, noise power and received signal power are estimated in the frequency domain at the receiver. In this research, the noise variance is estimated in null subcarriers and the received signal power is estimated in active (pilot and data) subcarriers. Received power at null subcarriers is estimated as

(17) $${\hat{\sigma }}_{n}^{2}=\frac{1}{{\bar{K}}_{n}}\overset{{\bar{K}}_{n}}{\underset{n=1}{\sum }}{\left|r_{f}\left[K_{n}\left(n\right)\right]\right|}^{2}$$

where $K_{n}$ is the set containing the indices of null subcarriers as the set $K_{n}=\left\{n_{1},\ldots ,\;n_{K_{n}}\right\}$.

Received power at active subcarriers is estimated as

(18) $${\hat{\sigma }}_{a}^{2}=\frac{1}{{\bar{K}}_{a}}\overset{{\bar{K}}_{a}}{\underset{a=1}{\sum }}{\left|r_{f}\left[K_{a}\left(a\right)\right]\right|}^{2}$$

where $K_{a}$ is the set containing the indices of pilot and data subcarriers, i.e., $K_{a}=K_{p}\cup K_{d}$ as the set $K_{a}=\left\{a_{1},\ldots ,\;a_{K_{a}}\right\}$. Then, the average SNR in the frequency domain can be estimated as [5]

(19) $$\bar{\gamma }=\frac{{\hat{\sigma }}_{a}^{2}}{{\hat{\sigma }}_{n}^{2}}-1$$

## 4. Adaptive Modulation

Limited bandwidth and time-varying multipath propagation cause significant constraints on the attainable throughput of UA communication systems. Therefore, adaptive OFDM modulation schemes have been introduced to improve the bandwidth efficiency and data rate of UA communication systems. In adaptive OFDM modulation, each subcarrier can be independently modulated, or all subcarriers can be modulated in the same manner [26]. In this research, frame-based and cluster-based adaptive modulation schemes have been developed, where the adaptation is performed frame by frame. In a frame-based adaptive scheme, all subcarriers of one frame are modulated with one modulation size, and in a cluster-based adaptive scheme, group of subcarriers (clusters) of one frame are modulated with different modulation sizes. 

**Figure 2:**
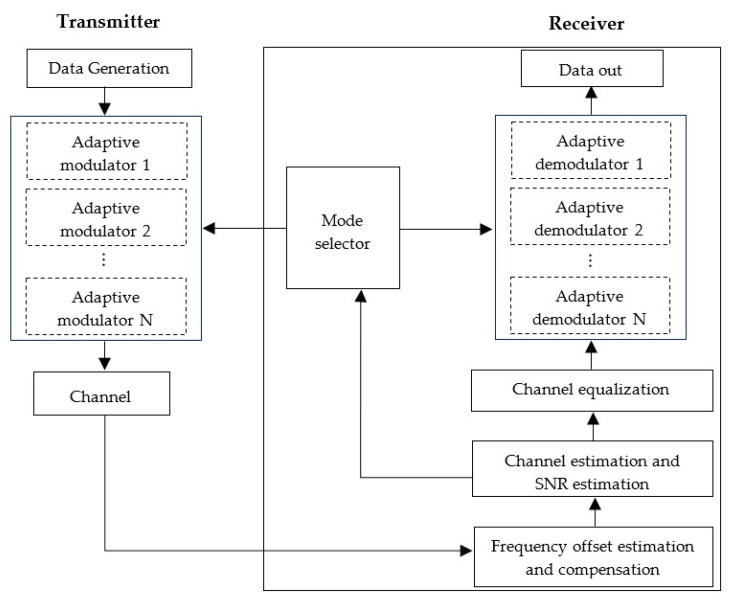
Block diagram of adaptive modulation scheme.

The block diagram of the proposed adaptive modulation scheme is shown in Figure 2. The adaptive modulator/demodulator blocks at the transmitter/receiver side consist of different modulators/demodulators, which provide different modulation/demodulation mode. The channel estimation and SNR estimation are performed after the frequency offset estimation and compensation at the receiver side. Then, depending on the estimated received SNR (frame-based)/received cluster SNR (cluster-based), the modulation modes are selected by the mode selector block for the next frame (frame-based)/each cluster of the next frame (cluster-based), which are fed back to the transmitter. Then, the adaptive modulator block modulates the data frame (frame-based)/clusters of the data frame (cluster-based), and the demodulator block demodulates the received signal accordingly.

The estimated received SNR and received cluster SNR are used as the CSI for the frame-based and the cluster-based adaptive modulation system, respectively. Before performing adaptive modulation, fixed modulation for each individual modulation scheme (i.e., BPSK, QPSK, and 16QAM) is performed. The SNR is estimated at the receiver side and the corresponding BER is calculated. Then, the SNR versus BER performance results of the fixed modulation for each individual modulation scheme are plotted in a graph. After investigating the plotted results, the target BER and SNR switching thresholds are determined to perform the adaptive modulation. The transmission parameter selections are subject to estimated SNR and SNR thresholds. This process is also clearly discussed in Section 6 with the BER performance graph and SNR thresholds table.

### 4.1. Frame-Based Adaptive Modulation

In a frame-based adaptive modulation scheme, the adaptation is performed frame by frame. The estimated received SNR is used as CSI to select the modulation size, depending on SNR thresholds for the next transmission. Then, the information containing modulation size is fed back to the transmitter for the next data frame. Then all the data subcarriers of the next data frame are modulated with the same modulation size. Depending on the channel conditions, the frame-based scheme permits to choose the highest-order modulation which allows to send more bits per symbol, and thus higher data rate and better spectral efficiency can be achieved. Compared with cluster-based adaptive modulation, the frame-based adaptive modulation requires less amount of CSI feedback, as only one modulation size needs to feedback for each frame for the next transmission.

### 4.2. Cluster-Based Adaptive Modulation

In the frame-based adaptive scheme, all subcarriers in a frame are modulated in the same manner, i.e., with the same modulation size. On the other hand, in the cluster-based adaptive modulation, clusters (group of subcarriers) in one frame are modulated with different modulation size, depending on the received cluster SNR. 

A cluster consists of a group of adjacent subcarriers. When the channel changes slowly across frequency, neighbouring subcarriers have similar SNR. Hence, the same modulation size is allocated for the neighbouring subcarriers. In such instance, feeding back transmission parameters for each subcarrier is not necessary. The feedback can be performed for the group of subcarriers (cluster), i.e., the total number of bits that are fed back to the transmitter can be reduced, resulting in a reduction in the computational load of the system [9,41]. For this reason, cluster-based adaptive modulation is studied in this section.

One of the factors that deteriorates the BER performance of UA systems is the deep-fading subcarriers of the UA channel. As a result, a large amount of transmitting energy is wasted on the deep-fading subcarriers [42]. In the cluster-based adaptive modulation scheme, depending on the received cluster SNR, the deep-faded subcarriers (weak subcarriers) are discarded for the next transmission. That is how the subcarriers are adaptively chosen by the system.

In contrast to the frame-based adaptive modulation scheme, the received cluster SNR of each cluster of the current frame is used as CSI for choosing the transmission parameters for the next frame in the cluster-based adaptive modulation scheme. The clusters that are in a deep fade are discarded from the next transmission, which means no data are transmitted and zero power is allocated for those clusters. Then, residual power is distributed among the remaining clusters and data are transmitted through the remaining clusters with proper modulation size depending on the CSI (cluster received SNR). That is how the modulation sizes are adaptively chosen and how residual power is adaptively distributed by the system.

**Figure 3:**
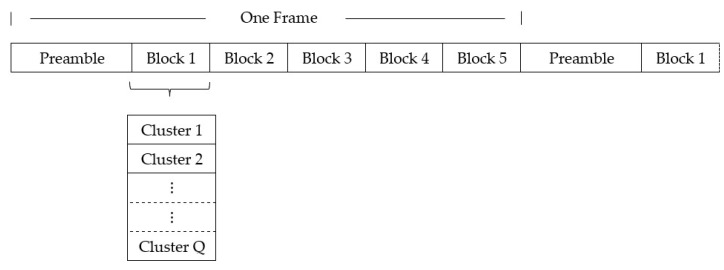
Cluster structure in UA OFDM frame.

This adaptive modulation scheme is called cluster-based adaptive modulation, as the subcarriers in each block of the frame are divided into group of subcarriers (cluster) to reduce the computational complexity and feedback load. Each cluster contains combination of pilot and data subcarriers (active subcarriers). Figure 3 shows the cluster structure in a frame.

The active subcarriers are $K_{a}$, where $K_{a}=K_{p}\cup K_{d}$ [5] are grouped into Q clusters. The size of each cluster in each OFDM block is

$$C={\bar{K}}_{a}/Q.$$

**Figure 4:**
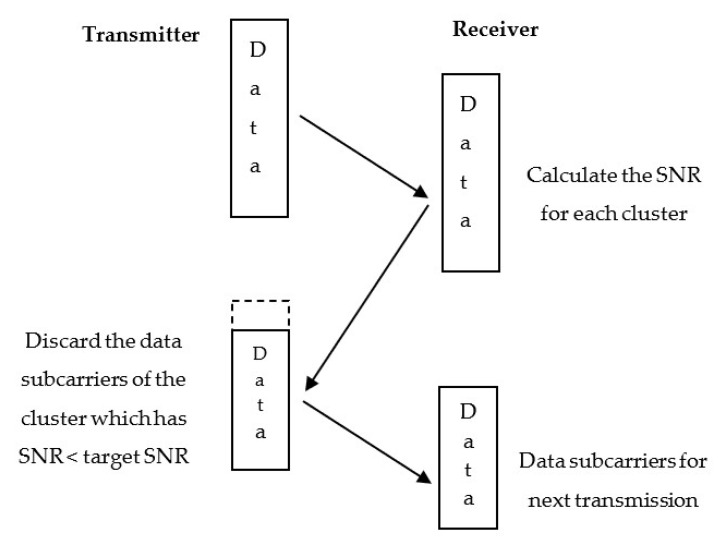
Discarding data subcarriers depending on the cluster SNR estimation.

The SNR of each cluster in the frequency domain can be estimated as

(20) $$\bar{\gamma_{q}}=\frac{\frac{1}{{\bar{K}}_{a_{q}}}\sum_{m_{q}=1}^{{\bar{K}}_{a_{q}}}{\left|r_{f}\left[K_{a_{q}}\left(m_{q}\right)\right]\right|}^{2}}{\frac{1}{{\bar{K}}_{n}}\sum_{m=1}^{{\bar{K}}_{n}}{\left|r_{f}\left[K_{n}\left(m\right)\right]\right|}^{2}}-1$$

where $K_{a_{q}}$ is the set containing the indices of pilot and data subcarriers of the qth cluster. In the cluster-based adaptive modulation scheme, after estimating the received cluster SNR using Equation (20), an algorithm is followed to discard and keep the clusters for the next transmission, which is shown in Figure 4. If the received SNR of a cluster is less than the target SNR, then the data subcarriers of this cluster are discarded from the next transmitted frame. If the received SNR of a cluster is greater than the target SNR, the data subcarriers of this cluster are kept for the next transmission. This information is sent back to the transmitter along with the modulation size for each remaining clusters for the next transmission.

If the received cluster SNR is less than the target SNR, the BER of that cluster is high; therefore, it is ineffective to send information data through weak clusters. Moreover, by discarding the weak clusters, the system can save energy which is distributed among the remaining clusters, and make the system energy efficient. Hence, if the discarded cluster is large, the residual power distribution helps to keep the communication quality unaffected. The weak clusters can be resumed in the next transmission if the channel condition improves.

After discarding the data subcarriers under a target SNR, the remaining OFDM data symbols in the next frame are generated as

(21) $$s_{remd}={\left(s_{d}\left[1\right],\ldots ,s_{d}\left[\;K_{remd}\right]\right)}^{T}$$

where $K_{remd}$ is the number of remaining data subcarriers in the next frame. The total power of the OFDM symbol is

(22) $$P_{total}=P_{remd}+P_{res}$$

where $P_{remd}$ is the power of the remaining data subcarriers and $P_{res}$ is the power of the discarded subcarriers of the current frame.

As the data subcarriers under the target SNR are discarded, the total power of the data subcarriers is reduced for the next frame. To further improve the system performance, the residual power $P_{res}$ is distributed among the remaining subcarriers for the next frame. After distributing $P_{res}$ in the remaining subcarriers, the data symbols of the next frame are represented as

(23) $$s_{d}=\beta {\left(s_{d}\left[1\right],\ldots ,s_{d}\left[K_{remd}\right]\right)}^{T}$$

where $\beta =\sqrt{K_{d}/K_{remd}}$ is the distribution of average power of the data subcarriers.

In this research, modulation size is fed back from the feedback link transmitter to the feedback link receiver. Receiving the right modulation size for the frames (frame-based) and clusters (cluster-based) is important. In frame-based adaptive modulation, only one modulation size is fed back for the next frame, whereas in cluster-based adaptive modulation, Q modulation sizes need to be fed back for Q clusters of a frame. A successful modulation/demodulation process of the forward channel entirely depends on the correct detection of modulation size at the feedback channel. To make the feedback link reliable and for errorless detection of the bits containing modulation size, repetition code is considered at the feedback transmitter, and majority voting is considered at the feedback receiver. At the feedback transmitter, bits are repeated several times for each modulation size and the bits are recovered at the feedback receiver by searching the bit stream that occurs most often.

## 5. System Implementation

### 5.1. System Hardware

In our adaptive UA OFDM communication system, CompactDAQ, a data acquisition platform, is adopted which is manufactured by NI. CompactDAQ system integrates hardware for data I/O with NI LabVIEW software for collecting, processing, and analysing sensor data. It comprises a plug-and-play chassis and NI I/O modules. The chassis is designed for small portable sensor measurement systems which control the synchronization, timing, and data transfer among I/O modules and an external host (computer) via a USB cable. In the proposed system, a four-slot NI cDAQ-9174 plug-and-play chassis, two-channel NI 9260 module, and a three-channel NI-9232 module are used. The NI 9260 module is used for data generation and the NI-9232 module is used for data acquisition. These two NI I/O modules are plugged into two of the four slots of the chassis. Then, the NI cDAQ-9174 chassis is connected to a laptop through USB, where the NI LabVIEW software is installed for the signal generation, acquisition, and processing. The transducer is connected to the NI-9260 module through a transformer-matching network and a power amplifier to transmit acoustic signals through the UA channel. The hydrophone is connected to the NI-9232 module through preamplifier to acquire signals received from the UA channel. Two sets of devices are employed for performing adaptive modulation. One set is designed for the forward link, and the other set is designed for the feedback link. 

### 5.2. Software Implementation

NI LabVIEW software is considered for designing the software of the proposed adaptive UA OFDM communication system. The transmitter generates one frame in LabVIEW and forwards the generated data frame to channel 1 of the data-generation module NI 9260 (DAQ1/Slot2/channel1) where the transducer (a) is connected. The transducer (a) transmits the signal through the UA forward channel. Then, the hydrophone (a) receives the signal and forwards the received data to channel 1 of the data acquisition module NI9232 (DAQ2/Slot1/channel1). Then, the receiver in LabVIEW starts to process the received signal samples by converting from the passband to the baseband. Then, after removing the CP from each OFDM block, the frequency offset estimation and compensation for each OFDM symbol are performed. Then, the channel estimation and SNR estimation are performed in the frequency domain. Using the estimated channel response, the received signals are equalized, and then the demodulation operation is performed to the equalized signals.

**Figure 5:**
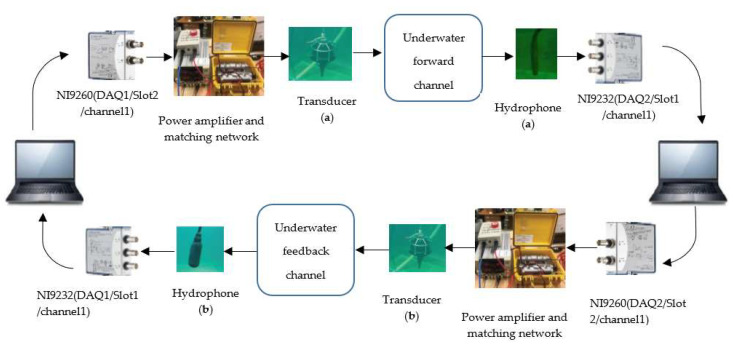
LabVIEW-based implementation of real-time adaptive modulation for UA OFDM system.

Depending on the SNR estimation, the transmission parameters are adaptively selected. For performing the adaptive modulation scheme, data symbols containing the adaptively selected transmission parameters information are modulated by QPSK constellations at the feedback transmitter in LabVIEW. Then, the data frame is forwarded to channel 1 of the data generation module NI-9260 (DAQ2/Slot2/channel 1) of the feedback link. Then, the transducer (b) feeds back the transmission parameters information signal through the UA feedback channel. The hydrophone (b) receives the signal and forwards the received data containing transmission parameters to channel 1 of the data acquisition module NI9232 (DAQ1/Slot1/channel1) of the feedback link for processing. Figure 5 shows the real-time adaptive modulation scheme for UA communication.

## 6. Results and Discussion

### 6.1. Frame-Based Adaptive Modulation

At first, extensive performance results of each individual modulation scheme (i.e., BPSK, QPSK, and 16QAM) have been observed with fixed modulation for UA communication systems for the frame-based adaptive modulation scheme. After investigating the fixed modulation results, the target BER and the switching thresholds for performing adaptive modulation are determined. Switching threshold keeps the overall BER of the system lower than the target BER. In this scheme, the estimated received SNR is used as switching parameter [8,43]. This adaptive scheme allows to choose the highest modulation size under a certain BER and SNR. Consequently, a better trade-off between data rate and overall BER is achieved by the proposed adaptation system.

The effect of Doppler frequency in adaptive modulation for UA OFDM system is also investigated in the frame-based adaptive scheme. Doppler frequency results in significant ICI. Due to ICI, the power of received signal in the inactive (null) subcarriers enhances, and the detection of transmitted signal on active subcarriers misleads. Hence, the accuracy of the SNR estimation is affected, which in turn significantly degrades the performance of the adaptive modulation.

#### 6.1.1. Simulation Results

**Figure 6:**
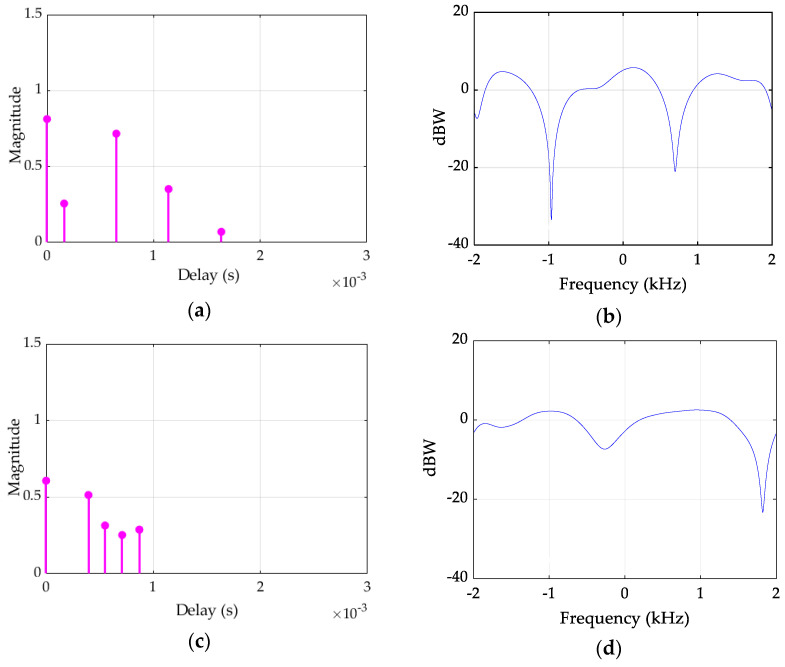
CIRs and CFRs of the UA channel for the first OFDM frame (**a**,**b**) and the fifth OFDM frame (**c**,**d**).

Randomly time-varying multipath propagation characteristic of UA channels results in frequency selective fading [40]. Furthermore, Doppler shift is introduced to the channel due to motion of the transmitter and/or receiver which contributes to the changes in CIR. Figure 6 depicts the simulation results of the time-varying CIRs and CFRs of the first and the fifth OFDM frames when the motion speed is 0.01 m/s. At each path, the channel gain is Rayleigh distributed. From Figure 6a,c, it is observed that there are five paths in the channel, and the arrival time (delay (s)) of the five paths are different between the first and the fifth OFDM frames. It is also seen that the magnitude of CIRs is varying fast between the first and the fifth frame. In Figure 6a,c, both the arrival time and magnitude of the paths are varying, which illustrate the time-varying characteristics of the multipath channel. Figure 6b,d show the frequency response of the channel. The frequency selective fading is also observed from Figure 6b,d as the power level (dbW) varies over the signal bandwidth.

**Figure 7:**
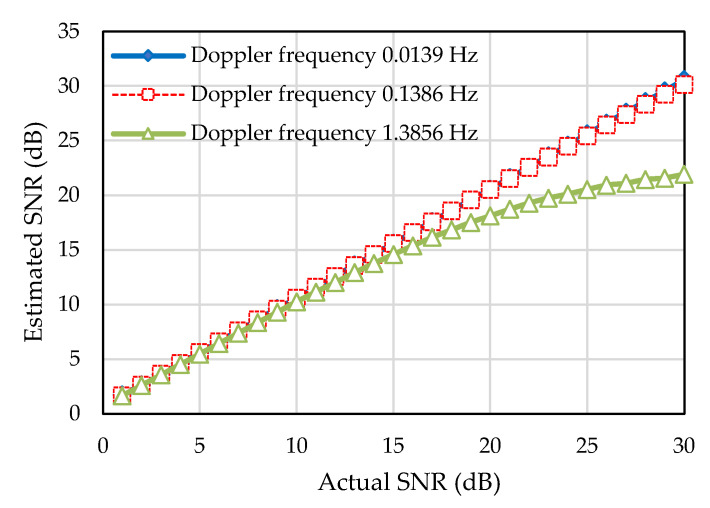
Estimated SNR for different Doppler frequencies for fixed modulation.

In this research, the performance of the proposed adaptive modulation relies on the accuracy of the SNR estimation, as the received SNR is determined as switching parameter. The estimated SNR needs to be as close as possible to the actual SNR. At lower Doppler frequency, the SNR estimation algorithm can estimate the SNR nearly close to the actual SNR. However, at higher Doppler frequency, the SNR is not estimated close to actual SNR as the estimated noise power increases due to ICI. Figure 7 shows the estimated SNR for different Doppler frequencies for fixed modulation. The estimated SNRs for lower Doppler frequency of 0.0139 Hz and 0.1386 Hz are depicted by the diagonal upward straight line, which indicates that the estimated SNR is close to the actual SNR at lower Doppler frequency. However, at higher Doppler frequency of 1.3856 Hz, the SNR is estimated close to the actual SNR up to 15 dB, then the diagonal straight line starts to bend downward when SNR reaches above 15 dB. Therefore, it can be said that adaptive modulation performs well around the lower SNR range (<15 dB) at higher Doppler frequency.

**Figure 8:**
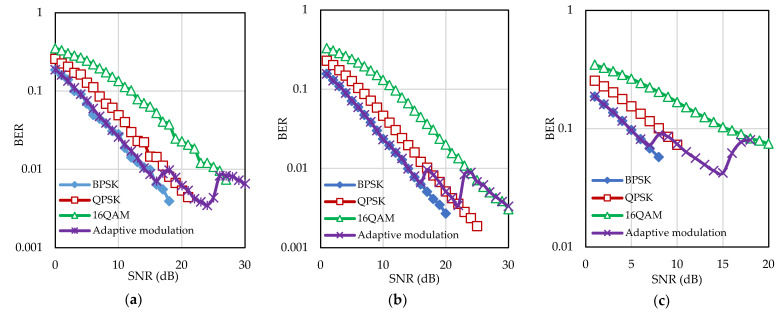
BER performance of the proposed adaptive modulation scheme for Doppler frequency (**a**) 0.0139 Hz, (**b**) 0.1386 Hz, and (**c**) 1.3856 Hz.

For simulation, 512 subcarriers are considered in each block of the UA OFDM system and five paths are considered in the UA channel. Maximum Doppler shift varies from 0.0139 Hz to 1.3856 Hz. At first, the BER performance of the fixed modulation using BPSK, QPSK, and 16QAM constellations are studied. Then, after investigating the SNR versus BER results of the fixed modulation, the target BER and switching thresholds are determined. Figure 8 shows the BER performance of adaptive modulation and fixed modulation system for different Doppler frequencies. It is seen that, for the lower Doppler frequency (0.0139 Hz and 0.1386 Hz), the target BER is selected as low as 0.01. On the other hand, for higher Doppler frequency (1.3856 Hz), the selected target BER is 0.1. Switching thresholds used in the simulation for the frame-based adaptive modulation schemes are presented in Table 1 for Doppler frequencies of 0.0139 Hz, 0.1386 Hz, and 1.3856 Hz. The target BER is higher for higher Doppler frequency compared to lower Doppler frequency, as Doppler frequency-induced ICI places a limit on the SNR estimation at higher SNR range. Therefore, it can be said that in the presence of ICI, not only the actual noise power but also the ICI is reflected in the estimated noise power which affects the SNR estimation. This means that the performance of the proposed adaptive modulation scheme becomes limited not only by noise but also interference at higher Doppler frequency. 

In simulation, the preamble block contains $K_{pre}=510$ subcarriers. In data blocks, among the total 512 subcarriers, there are 320 data subcarriers, 128 uniformly spaced pilot subcarriers, and 64 null subcarriers. The number of information-carrying uncoded bits in each frame is $K_{b}=1600$ for BPSK, $K_{b}=3200$ for QPSK, and $K_{b}=6400$ for 16QAM. The data rate of the fixed modulation system is estimated as

(24) $$D_{r}=\frac{K_{b}}{T_{fr}}$$

where frame duration is $T_{fr}=\left(\left(T+T_{cp}\right)K_{blk}\right)+T_{pre}$, and $T_{pre}$ is the preamble duration.

**Figure 9:**
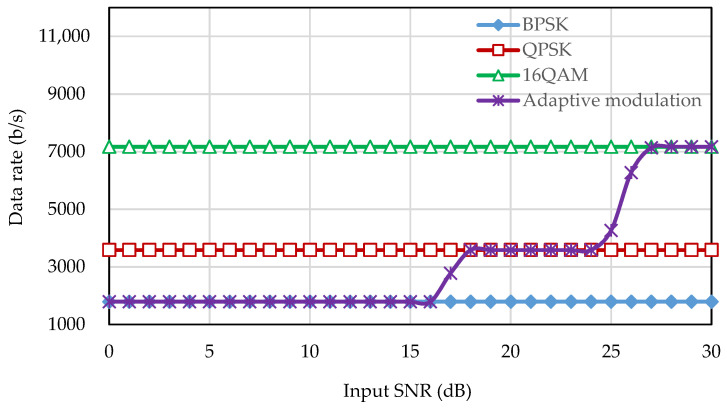
Data rate of the frame-based adaptive modulation.

The data rate of fixed modulation is fixed for each transmission. In this simulation, the data rates of BPSK, QPSK, and 16QAM are 1.79 kbps, 3.59 kbps, and 7.17 kbps, respectively. In contrast, the data rate of adaptive modulation changes in each transmission depending on the estimated received SNR. The data rate of the fixed modulation and the adaptive modulation is shown in Figure 9. It can be seen that the data rate of the fixed modulation is constant at any channel condition whereas the data rate of the adaptive modulation changes according to the channel conditions. From the simulation results, it can be said that no fixed OFDM system can provide a better BER performance while simultaneously providing a better data rate. The proposed adaptive system can ensure a higher data rate at the target BER compared with the fixed modulation.

#### 6.1.2. Experimental Results

**Figure 10:**
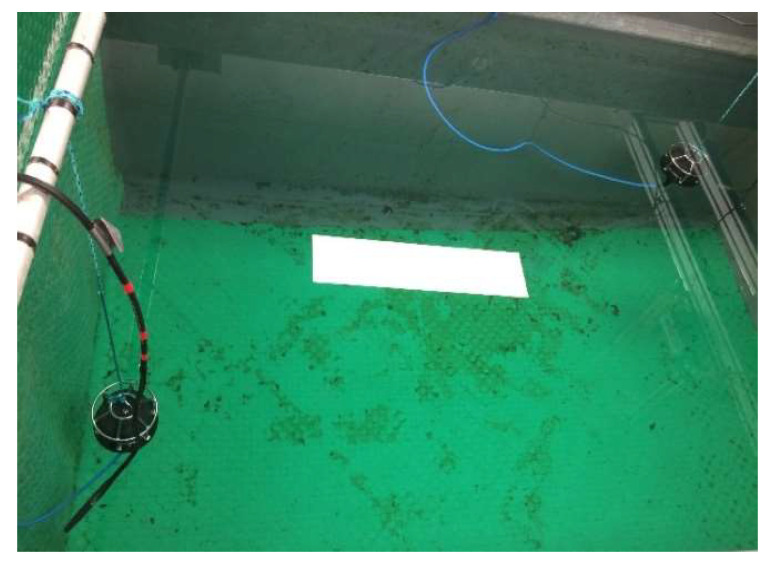
Location of the transducers and hydrophones for the forward and feedback links during the frame-based adaptive modulation tank experiment.

In the tank experiment, two CTG0052 transducers, one Reson reference hydrophone (forward link) and one HTI-96-Min hydrophone (feedback link) were used. At first, a pair of transducer and hydrophone is used to perform a one-way communication experiment for fixed modulation using BPSK, QPSK, and 16QAM constellations. During the experiment, the received SNR is altered through varying the power of the transmitted signal. By plotting the average received frame SNR vs. BER results of the fixed modulation system, the SNR thresholds are chosen. After the SNR thresholds are obtained, another pair of transducer and hydrophone is placed in the tank to perform the experiment of adaptive modulation. Figure 10 shows the location of the transducers and hydrophones in the tank during the experiment. The length, width, and depth of the tank are 2.5 m, 1.5 m, and 1.8 m, respectively. During the experiment, the frequency offset estimation and compensation module in Figure 2 is bypassed as the Doppler shift is very small in a tank. The system parameters used for the adaptive modulation schemes in the tank experiment are listed in Table 2.

**Figure 11:**
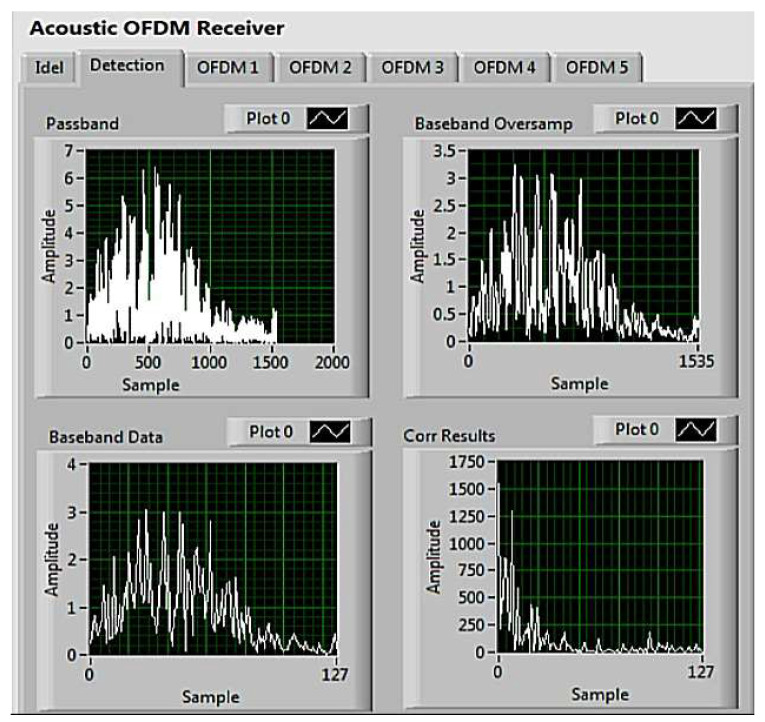
Received data frame in acoustic OFDM receiver during the tank experiment.

**Figure 12:**
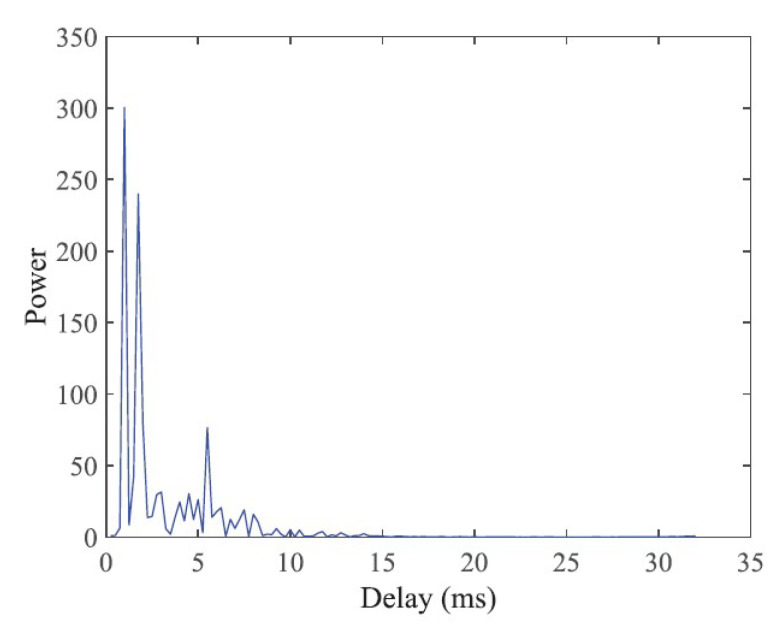
Estimated power of the CIR during the tank experiment.

After transmitting the signal through the UA channel, a successfully detected data frame and magnitude of the cross-correlation between the local synchronization sequence and received synchronization sequence are shown in Figure 11. The peak position indicates the strongest channel path position of the UA channel while other non-negligible local peak values represent the positions of the other channel paths. This proves that there are multiple paths between the transmitter and receiver resulting from the reflections of acoustic signals from the wall of the tank. Figure 12 shows the power of the CIR estimated by the pilot subcarriers. It can be seen that the maximum channel delay spread is 15 ms, which is shorter than the CP length.

**Figure 13:**
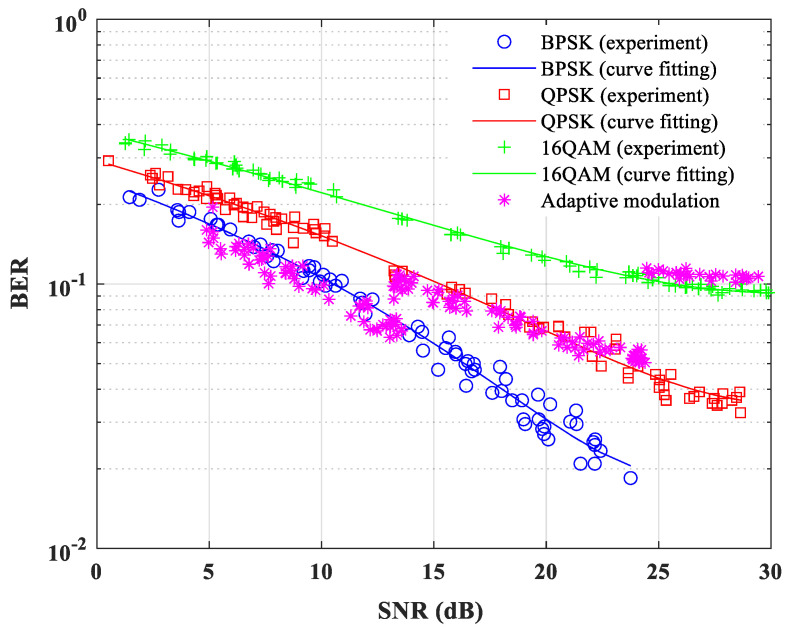
BER performance of the proposed frame-based adaptive modulation schemes in tank experiment.

The BER vs. SNR results are shown in Figure 13 for fixed modulation and adaptive modulation schemes. Using the BER performance results of the fixed modulation based on BPSK, QPSK, and 16QAM constellations, the target BER and the switching thresholds are determined for the adaptive modulation scheme to keep the overall BER lower than the target BER. The uncoded target BER is chosen as 0.1 for this experiment, and the switching thresholds are presented in Table 3.

### 6.2. Cluster-Based Adaptive Modulation

In the cluster-based adaptive modulation scheme, data subcarriers are grouped in 22 clusters and the SNR is estimated for each cluster. Depending on the estimated received cluster SNR, the data subcarriers of the weak clusters are discarded and the remaining clusters, i.e., data subcarriers, are selected for the next transmission with modulation sizes depending on the channel condition. Then, the residual power is distributed among the remaining data subcarriers for the next transmission. Thus, the cluster-based adaptive modulation scheme adaptively chooses the subcarriers and modulation size and allocates the power. 

#### 6.2.1. Experimental Results

The performance of the proposed cluster-based adaptive modulation scheme is verified through recent UA OFDM communication experiments performed in the Canning River, Western Australia, and the experimental results verify the superiority of the proposed adaptive scheme. The system parameters used during the experiment are listed in Table 4.

**Figure 14:**
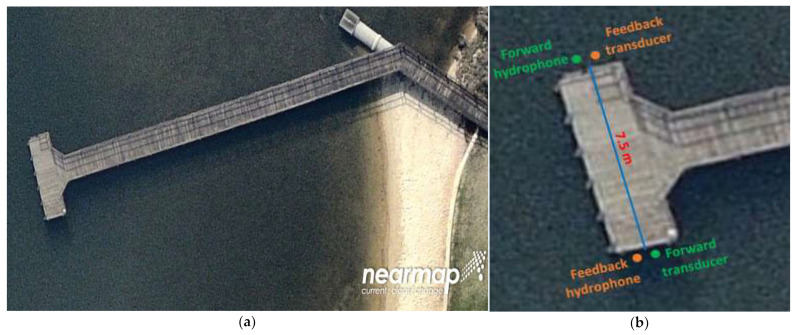
(**a**) The jetty in the Canning River where the real-time river experiment was carried out. (**b**) Location of the transducers and hydrophones in the river experiment for the cluster-based adaptive modulation.

Figure 14a shows the jetty in the Canning River where the real-time river experiment was carried out. Figure 14b shows the location of the transducers and hydrophones in the river during the cluster-based adaptive modulation experiment. The distance between the forward transducer and feedback transducer was around 7.5 m. The water depth along the direct path varied between 1 m and 1.2 m. Two CTG0052 transducers and two Reson reference hydrophones were used for the river experiment.

**Figure 15:**
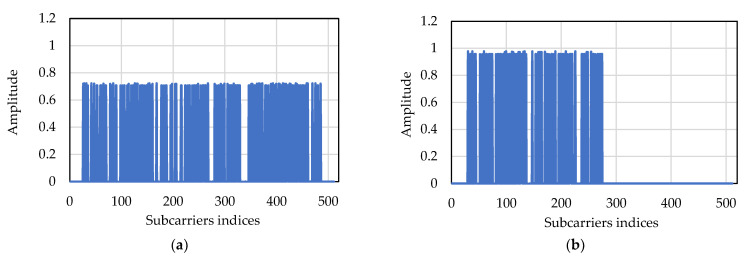
(**a**) Subcarrier amplitude when all clusters are active, and (**b**) subcarrier amplitude when 10 of the clusters are discarded.

During the river experiment, firstly, the power allocation algorithm was performed and verified as shown in Figure 15. Two frames were transmitted with modulation size 2 (QPSK). Figure 15a shows that the first frame was transmitted keeping all clusters (data subcarriers) active, and Figure 15b shows that the next frame was transmitted discarding almost half of the clusters (first 12 active clusters and last 10 discarded clusters). From Figure 15, it can be seen that the residual power of the discarded data subcarriers is distributed among the remaining first 12 active clusters (data subcarriers) for the next transmission.

**Figure 16:**
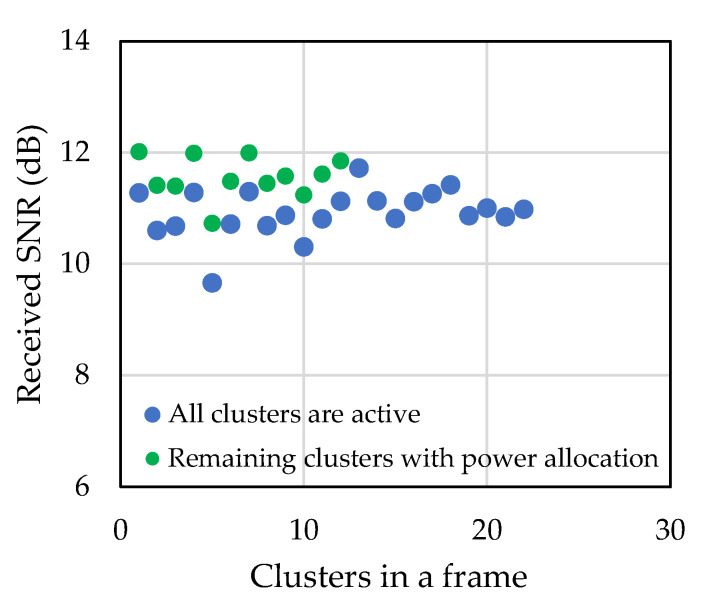
Estimated received SNR of the clusters of a frame.

**Figure 17:**
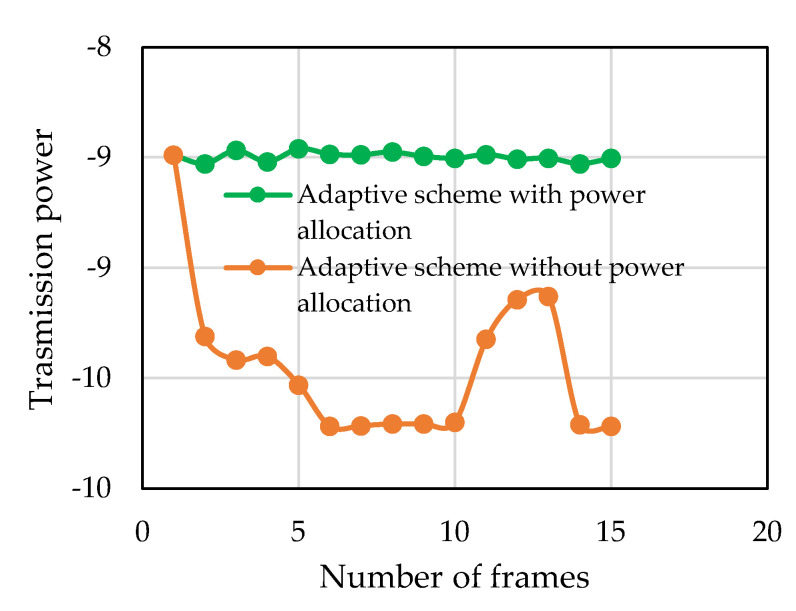
Transmitted symbol power for different channel conditions.

The distribution and allocation of the residual power among the remaining data subcarriers ensures constant transmitted symbol energy in spite of the channel variation. Figure 16 shows the estimated received SNR of the clusters in a frame. In the figure, the blue dots represent the SNRs when all the 22 clusters are active, i.e., all the data subcarriers are carrying information and the green dots represent the SNRs when the last 10 clusters were discarded and the first 12 clusters were carrying information. It can be seen that the estimated cluster SNR of the first 12 clusters is higher than the estimated cluster SNR when all the clusters were active, which also verified the power allocation to the remaining data subcarriers. Figure 17 shows the transmission symbol power with and without the adaptive power allocation algorithm during the river experiment. After discarding the deep-faded data subcarriers, if the residual power is not allocated among the remaining data subcarriers, then the transmission power of the next frames fluctuates, whereas if the residual power is allocated, then the transmission power always keeps almost constant. In summary, it can be said that when the channel condition is not good enough, the power allocation algorithm allocates more power to remaining active data subcarriers, which ensures overall better throughput of the system.

**Figure 18:**
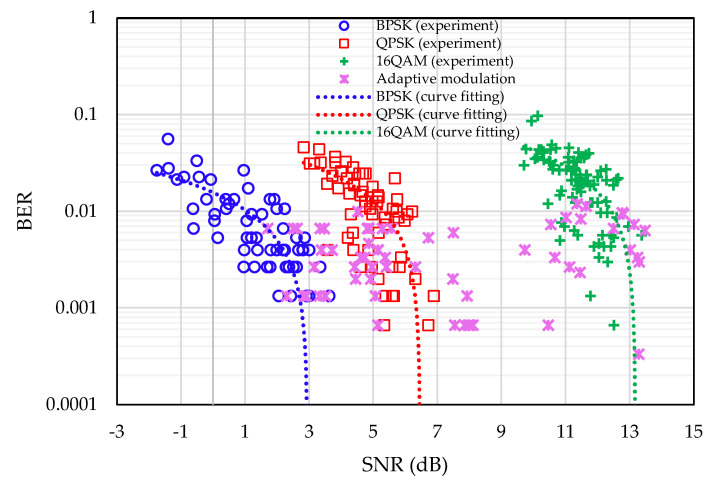
BER performance of the proposed cluster-based adaptive modulation scheme during the river experiment.

In the river experiment, fixed modulation is performed first for each individual modulation scheme (i.e., BPSK, QPSK, and 16QAM), and the results are plotted to determine the target BER and SNR thresholds. The BER performance results of the fixed modulation are shown in Figure 18. After investigating the results, the target uncoded BER is chosen as 0.01. Using the BER performance results of fixed modulation from Figure 18, the target SNR is selected to discard the deep-faded clusters, and a set of SNR thresholds are determined for switching the modulation mode for different remaining clusters. Switching thresholds for the cluster-based adaptive modulation scheme are presented in Table 5, which allows the system to keep the overall BER under the target BER. Figure 18 also shows the BER performance for adaptive modulation.

**Figure 19:**
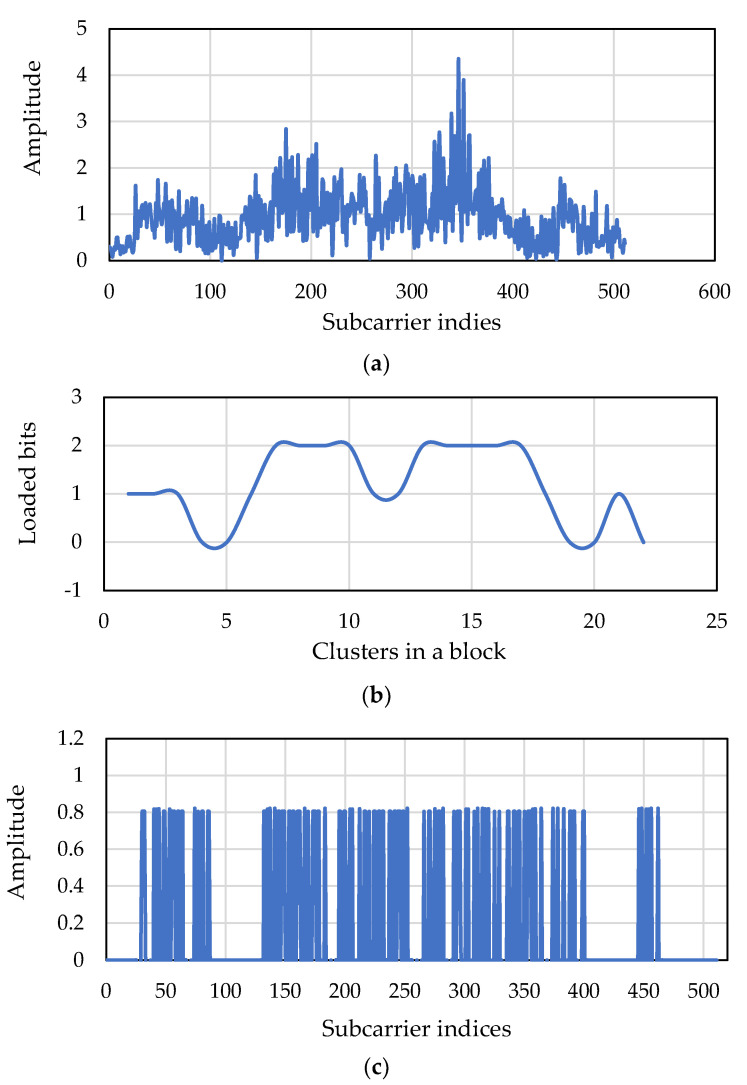
(**a**) CFR of the current OFDM block. (**b**) Loaded bits of the next OFDM block according to (**a**). (**c**) Power distribution for the data subcarriers of next OFDM block according to (**a**).

Figure 19a,b show the CFR of an OFDM block of a received frame and respective adaptively loaded bits in the clusters of that OFDM block for the next frame. It can be seen that the number of loaded bits, i.e., modulation size, for clusters are closely related to the CFR results. When the amplitude of the subcarrier cluster is higher, higher modulation size is selected, and vice versa. Figure 19c shows the power distribution for the data subcarriers of the next OFDM block accordingly.

**Figure 20:**
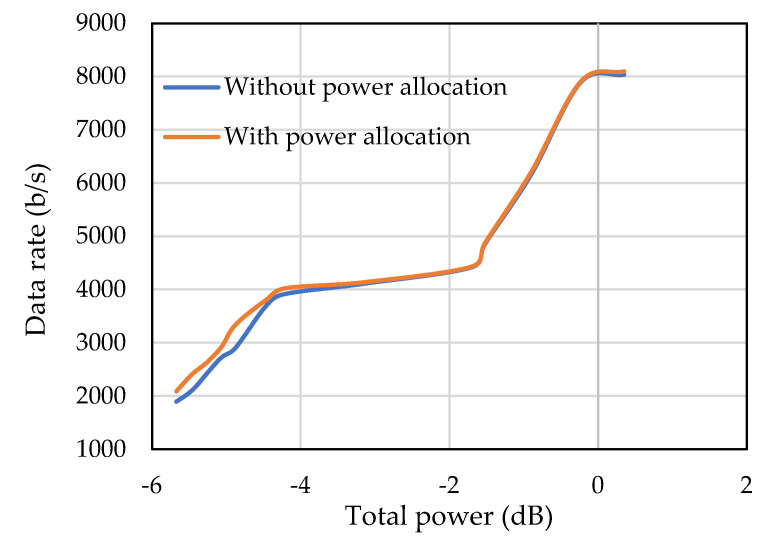
Data rate of the cluster-based adaptive modulation.

Figure 20 shows the data rate of the proposed cluster-based adaptive modulation scheme. It can be seen that the data rate also increases with the increase of the transmit power. When the transmit power is low, the deep-faded data subcarriers are discarded and data rate around the target SNR is lower, whereas the data rate is higher when the residual power is distributed among the remaining subcarriers. In summary, it can be said that the proposed cluster-based adaptive modulation adaptively allocates subcarriers, modulation size, and power, which improves the reliability of communication, reduces energy consumption, and ensures overall better throughput of the system.

## 7. Conclusions

In this paper, frame-based and cluster-based adaptive modulation schemes are proposed for UA OFDM communication systems. The estimated received SNR and received cluster SNR are used as CSI for frame-based and cluster-based adaptive modulation systems, respectively, and are used to choose the transmission parameters of the next transmission for performing the adaptive modulation. The consequences of Doppler frequency in adaptive modulation are also studied in this research. It is seen that Doppler-frequency-induced ICI affects the SNR estimation, which makes the adaptive modulation challenging. Therefore, it can be said that both the actual noise power and the ICI are reflected in the SNR estimation. Hence, estimated SNR is chosen as the performance metric for adaptive modulation for mode switching, which reflects the channel variation as well as Doppler effects.

In this paper, the performance results of the proposed schemes in the tank and the river experiments are also presented. Extensive fixed modulation systems based on BPSK, QPSK, and 16QAM schemes are investigated with both frame-based and cluster-based modulation schemes in UA OFDM systems to choose the target BER to perform adaptive modulation. In the frame-based adaptive modulation scheme, all subcarriers are modulated in the same manner. Hence, compared with the cluster-based scheme, it requires low feedback rate, which makes it more robust than the cluster-based scheme. A better trade-off between data rate and overall BER is attained in the frame-based adaptive modulation scheme, as the highest modulation order is chosen under a certain BER and SNR. 

In the cluster-based adaptive modulation scheme, subcarriers are grouped into clusters to reduce the computational and feedback load, and each cluster is independently modulated. Therefore, higher feedback rate is required. To make the feedback link reliable and for errorless detection of the bits containing modulation size, repetition code and majority voting are considered at the feedback link. The proposed cluster-based adaptive modulation scheme improves the system throughput under a fixed BER by choosing proper modulation sizes for clusters depending on the channel conditions, improves the energy efficiency of the system by discarding subcarriers that experience deep fade, and achieves overall better throughput of the system by distributing the residual power among the remaining subcarriers, which ensures constant transmitted symbol energy in spite of the channel variation. In summary, it can be said that the proposed cluster-based adaptive system is an energy-efficient and reliable communication system that improves the energy consumption of the system, ensures higher data rate, and guarantees continuous connectivity for a nonstationary time-varying UA channel.

## Figures and Tables

**Table 1:** Switching thresholds used in simulation for frame-based adaptive modulation schemes.

Mode	Modulation	Threshold for Doppler Frequency 0.0139 Hz	Threshold for Doppler Frequency 0.1386 Hz	Threshold for Doppler Frequency 1.3856 Hz
1	BPSK	SNR < 17.35 dB	SNR < 17.1 dB	SNR < 8 dB
2	QPSK	17.35 dB ≤ SNR ≤ 25.7 dB	17.1 dB ≤ SNR ≤ 23.4 dB	8 dB ≤ SNR ≤ 15.4 dB
3	16QAM	SNR > 25.7 dB	SNR > 23.4 dB	SNR > 15.4 dB

**Table 2:** Parameter used in frame-based adaptive modulation during tank experiment.

Bandwidth	B = 4 kHz
Number of subcarriers	$K_{s}$ = 512
Subcarrier spacing	$f_{sc}$ = 7.8 Hz
Length of OFDM symbol	T = 128 ms
Length of CP	$T_{cp}$ = 25 ms
Number of pilot subcarriers	$K_{p}$ = 128
Number of data subcarriers	$K_{d}$ = 325
Number of null subcarriers	$K_{n}$ = 59

**Table 3:** Switching threshold for adaptive modulation schemes in tank experiment.

Mode	Modulation	Threshold
1	BPSK	SNR < 13.25 dB
2	QPSK	13.25 dB ≤ SNR ≤ 24.5 dB
3	16QAM	SNR > 24.5 dB

**Table 4:** Parameters used in the cluster-based adaptive modulation during the river experiment.

Bandwidth	B = 4 kHz
Number of subcarriers	$K_{s}$ = 512
Subcarrier spacing	$f_{sc}$ = 7.8 Hz
Length of OFDM symbol	T = 128 ms
Length of CP	$T_{cp}$ = 25 ms
Number of pilot subcarriers	$K_{p}$ = 110
Number of data subcarriers	$K_{d}$ = 330
Number of null subcarriers	$K_{n}$ = 72

**Table 5:** Switching threshold for the cluster-based adaptive modulation in the river experiment.

Mode	Modulation	Threshold
1	Discard	SNR < 1 dB (target SNR)
2	BPSK	1 dB ≤ SNR < 5 dB
3	QPSK	5 dB ≤ SNR < 12 dB
4	16QAM	SNR ≥ 12 dB
